# Efficacy of Online Adaptive Radiotherapy Using Surface Guidance in Treatment of Tracheal Adenoid Cystic Carcinoma

**DOI:** 10.7759/cureus.67691

**Published:** 2024-08-24

**Authors:** Sanjana M Uppal, Sheereen Fatima, Prasad R Dandekar, Anand Jadhav, Sameer Pathan

**Affiliations:** 1 Department of Radiation Oncology, Sir H. N. Reliance Foundation Hospital and Research Centre, Mumbai, IND; 2 Department of Pathology, Sir H. N. Reliance Foundation Hospital and Research Centre, Mumbai, IND

**Keywords:** adenoid cystic carcinoma (acc), tracheal cancer, cone-beam computed tomography (cbct), surface-guided radiotherapy, online-adaptive radiotherapy

## Abstract

Primary tracheal tumors are rare, with adenoid cystic carcinoma (ACC) of the trachea being the second most common malignancy of the trachea. Radical surgical resection is found to have better survival outcomes in tracheal ACC. However, with higher submucosal spread rates in tracheal ACC and the inability to achieve clear margins, complete resection is not usually achievable. In these cases, the use of a 60-70 Gy radiation dose is deemed to be sufficient for definitive treatment with or without concurrent chemotherapy. We report a case of an unresectable ACC treated with online daily adaptive cone beam computed tomography (CBCT) radiotherapy on Ethos™ (Varian Medical Systems, Palo Alto, CA). She was planned to receive 59.4 Gy in 33 fractions in two phases. For daily treatment delivery, the patient was set up on the couch using the surface-guided radiotherapy (SGRT) system of AlignRT™ (Vision RT Ltd., London, UK) and translated to the treatment isocenter. A CBCT scan was acquired, followed by rigid registration with the planning scan and PET CT. Organs at risk (OAR) and primary targets were auto-generated by the AI in a two-step process, reviewed, and edited by the radiation oncologist. Adapted and scheduled plans were compared regarding planning target volume (PTV) coverage and dose to OAR. Better PTV coverage was seen in 26 of 33 fractions with the adapted plan. On the days with lesser coverage, adapted plans demonstrated improvement in the hotspot reduction and reduction in hard dose constraints of the esophagus and lungs. Hence, adapted plans were selected for all treatment days. Our results highlight the superior target coverage and improved OAR-sparing plans in daily online adaptive radiotherapy (o-ART) compared to image-guided radiotherapy (IGRT) plans. The system’s ability to adapt to daily anatomical changes, improved target coverage, and better sparing of OARs make it an encouraging option for malignancies requiring motion management.

## Introduction

Primary tracheal tumors are rare, with fewer than 0.2 cases reported per 100,000 people per year, and 10% of these are adenoid cystic carcinoma (ACC) of the trachea, making it the second most common malignancy of the trachea [1]. It is submucosal in origin, slow-growing, and demonstrates late distant metastasis [1,2]. Patients have a late clinical presentation due to the submucosal location of the tumor. The most common presentations are dyspnea, cough, wheezing, and hemoptysis [3]. Surgical excision is the mainstay of the treatment, and radiotherapy can be used as an adjuvant treatment [4]. Definitive radiotherapy is proposed for patients where the tumor is deemed unresectable or surgery is contraindicated [5]. We report a case of an unresectable ACC treated with online daily adaptive cone beam computed tomography (CBCT) radiotherapy on Ethos™ (Varian Medical Systems, Palo Alto, CA).

## Case presentation

A 43-year-old female, treated for tuberculosis 20 years ago, with no other comorbidities and a family history of breast cancer in a paternal cousin, was evaluated in May 2023 for recurrent cough, not associated with dyspnea or orthopnea. No significant findings demonstrating respiratory distress were elicited on general or systemic examination, and all routine baseline hematologic investigations were within the normal range.

**Figure 1:**
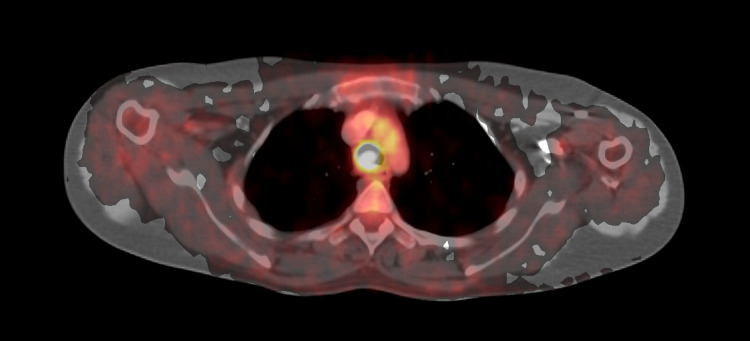
Ga-68 DOTATATE PET CT It shows a lesion of 1.3 × 1.6 × 1.5 cm with uptake of SUV max 6.3 and a minimal somatostatin receptor expression of SUV max 2.8.

**Figure 2:**
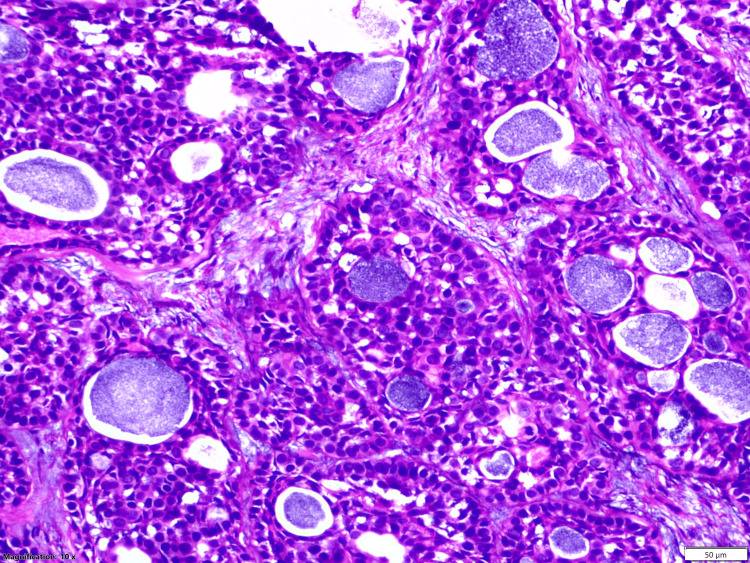
Histopathology Hematoxylin and eosin-stained tissue show biphasic tumors composed of nests, tubules, and cribriform structure of ductal and myoepithelial cells containing basophilic secretions scattered within a desmoplastic stroma at 50× magnification.

Further investigation with a video bronchoscopy revealed a tracheal growth that bled on touch. A contrast-enhanced CT scan of the chest showed an endoluminal polypoidal soft tissue density mass lesion arising from the right posterolateral wall of the trachea, 1.5 cm proximal to the carina at the D3-D4 vertebral level and causing almost 70% luminal compromise, without any obvious paratracheal extension. Ga-68 DOTATATE PET CT confirmed the above lesion of 1.3 × 1.6 × 1.5 cm with uptake of SUV max 6.3 and a minimal somatostatin receptor expression of SUV max 2.8, with no uptake elsewhere in the body (Figure 1). The patient was diagnosed as ACC of trachea on biopsy with ductal cells positive for CK7 and panCK, myoepithelial cells positive for p40 and calponin, and all cells negative for TTF-1, S100, chromogranin, and synaptophysin (Figure 2). Lung panel next-generation sequencing (NGS) detected a cKIT mutation in exon 11 and no other clinically relevant variants.

**Figure 3:**
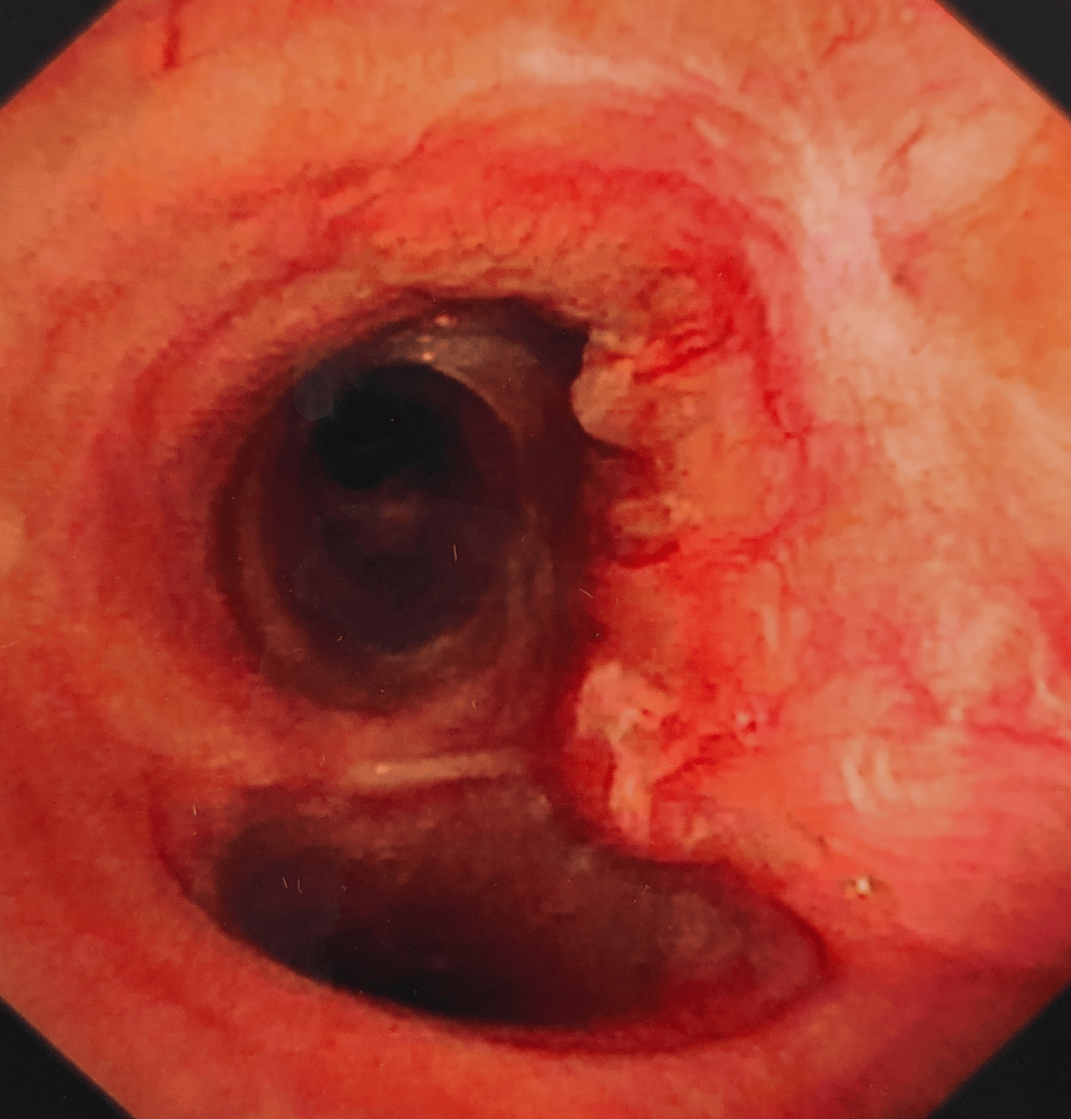
Bronchoscopy It showed a 5.5 cm long tumor on the posterior wall of the trachea with a luminal compromise of less than 10%.

The patient received four cycles of paclitaxel and carboplatin till October 2023. Follow-up PET CT revealed a stable plaque-like enhancing lesion measuring 1.2 × 0.5 cm of SUV max 3.2. Bronchoscopy showed a 5.5 cm long tumor on the posterior wall of the trachea extending up to the carina and on the right posterolateral wall just short of the right upper lobe bronchus, approximately 7 cm from the right vocal cord, spanning over six tracheal rings with a luminal compromise of less than 10% (Figure 3).

The patient had a static response to chemotherapy but still demonstrated a large volume of submucosal disease that was unresectable; hence, she was planned with local radiotherapy for disease control.

**Figure 4:**
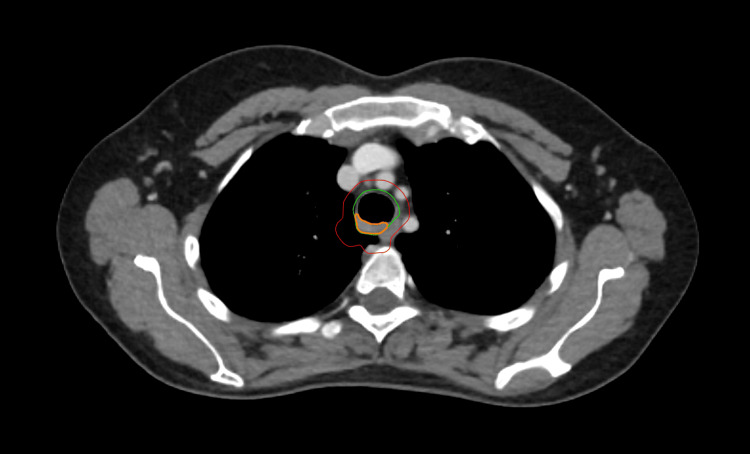
Contours for phase I Orange, gross tumor volume; green, clinical target volume; red, planning target volume

**Figure 5:**
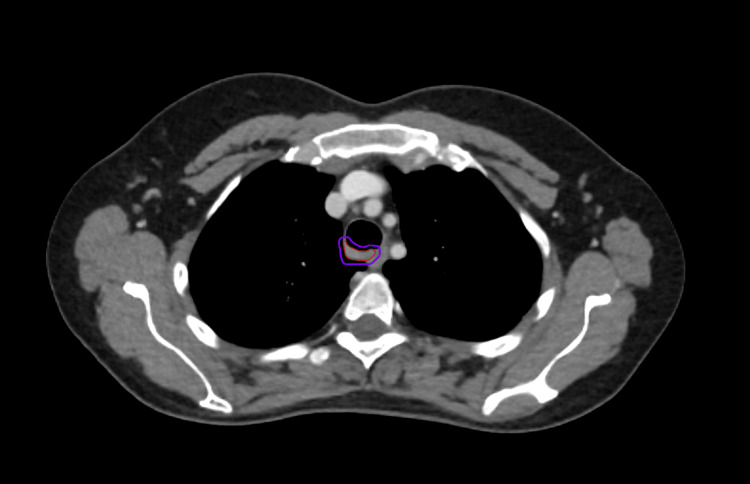
Contours for phase II Red, gross tumor volume; purple, planning target volume

**Figure 6:**
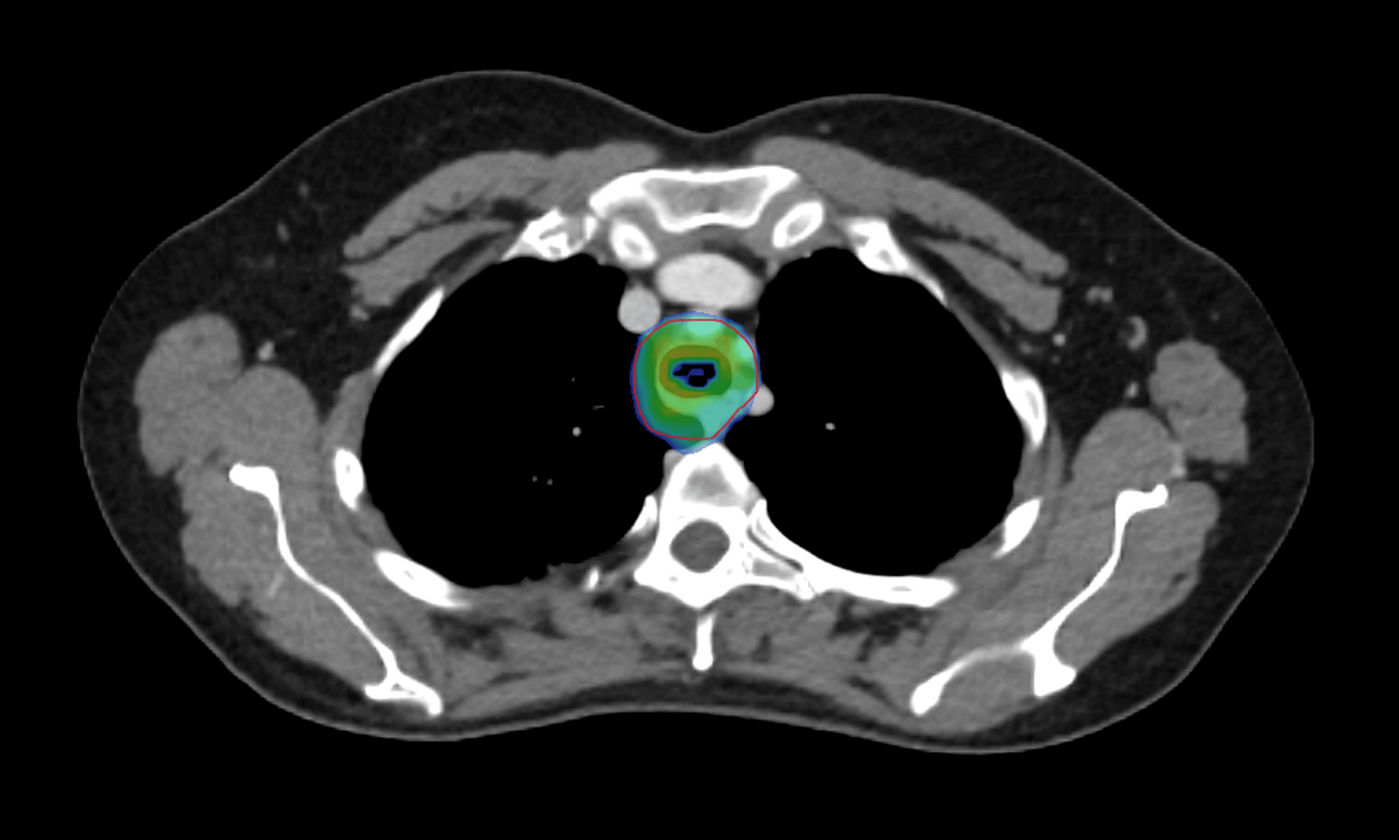
Dose wash for phase I It shows the 95% dose wash for phase I of treatment.

**Figure 7:**
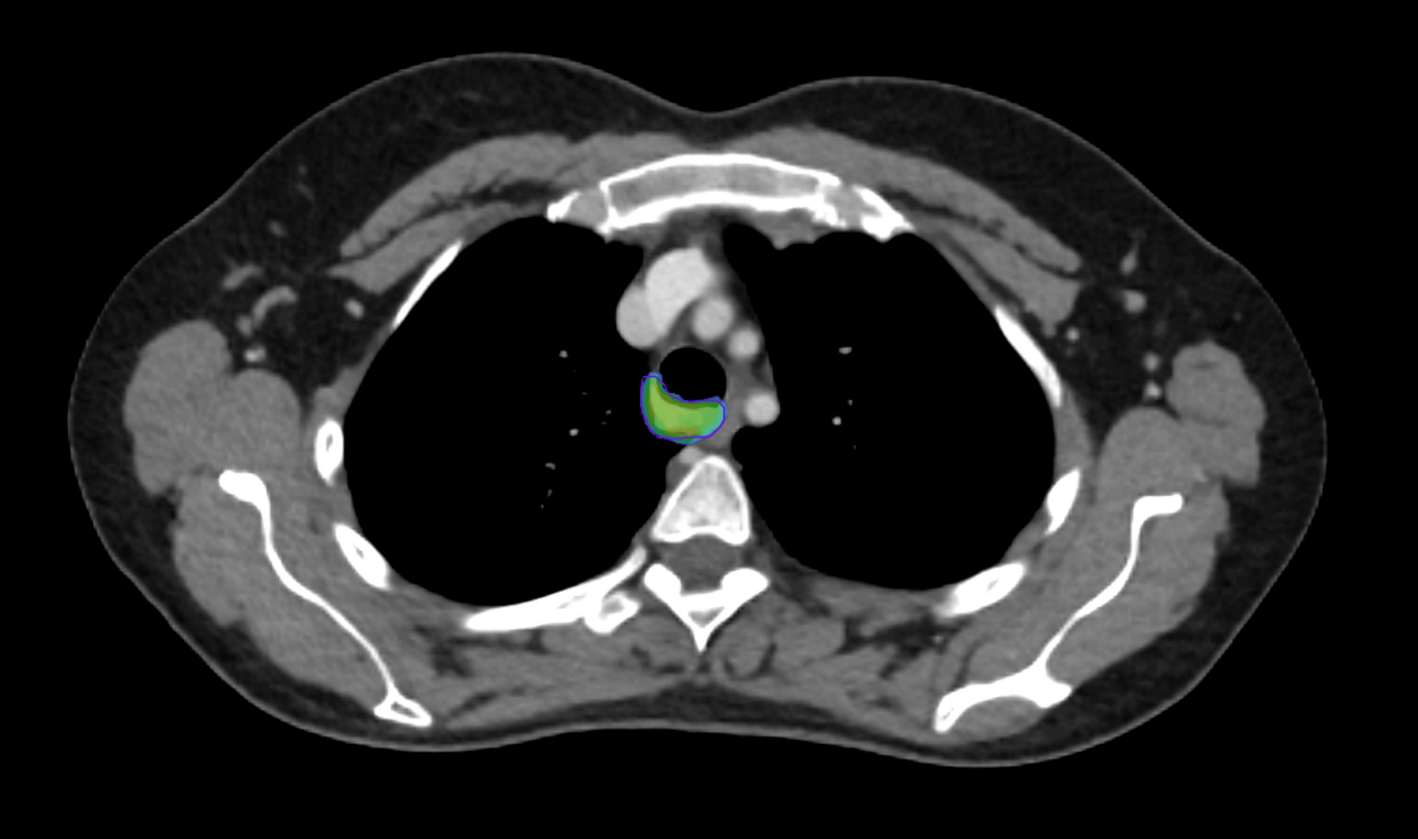
Dose wash for phase II It shows the 95% dose wash for phase II of treatment.

The patient was planned for treatment using the online adaptive radiotherapy (o-ART) technique of Ethos. She was planned to receive 59.4 Gy in 33 fractions in two phases. A custom-made immobilization device using a full body vac-lok with arms over the head and a small knee rest was made. A radiotherapy planning scan of 2 mm slice thickness was taken from the vertex to below the umbilicus. The gross tumor volume (GTV) was contoured on the planning scan after registration with a PET CT scan and correlating with the bronchoscopy report. This GTV was given a uniform 1 cm longitudinal and circumferential margin along with the inclusion of the entire tracheal circumference to generate the clinical target volume (CTV), editing out the esophagus and lungs. A 5 mm planning target volume (PTV) auto margin from this CTV was generated for the first phase of treatment to receive 54 Gy in 30 fractions (Figure 4). A direct 2 mm auto margin from GTV to PTV was generated for the second boost phase to receive 5.4 Gy in three fractions (Figure 5). The seven-field intensity-modulated radiotherapy (IMRT) plan was approved with 87.5% V95 coverage of PTV in phase I and 88.1% in phase II, with GTV coverage of 100% and 99.9%, respectively (Figures 6, 7). This lower numerical value of PTV coverage is because the Acuros algorithm of Ethos does not account for dose deposition in the air cavity of the trachea. The cumulative V20 of the lungs was 8.9%, and the Dmean of the esophagus was 24.59 Gy without any hot spot within it. All constraints achieved in the reference plan are elaborated in Table 1.

**Table 1:** Planning directives for reference plan GTV, gross tumor volume; PTV, planning tumor volume; V95%, volume receiving 95% of the dose; Dmax, maximum point dose; Dmean, mean dose; V3000 cGy, volume receiving 3000 cGy dose

Structure	Clinical goal	Priority	Reference plan
GTV_Phase I 54 Gy	V95% ≥ 95%	1	100%
GTV_Phase II 5.4 Gy	V95% ≥ 95%	1	99.9%
PTV_Phase I 54 Gy	V95% ≥ 95%	1	87.5%
	Dmax ≤ 105%	1	0%
PTV_Phase II 5.4 Gy	V95% ≥ 98-95%	1	88.1%
	Dmax ≤ 105%	1	0%
Both lungs	V2000 cGy < 20%	2	8.6%
	V500 cGy <50%	2	34.8%
	Dmean ≤ 2000 cGy	2	648 cGy
Esophagus	Dmean ≤ 3400 cGy	3	2459 cGy
	Phase I Dmax < 5400 cGy	1	5396 cGy
	Phase II Dmax < 540 cGy	4	531 cGy
Spinal canal	Dmax < 2500 cGy	2	2170 cGy
Heart	Dmean ≤ 3000 cGy	2	0%
	V3000 cGy < 30%	2	67 cGy
Larynx	Dmean ≤ 4400 cGy	3	122 cGy
Thyroid gland	Dmean ≤ 3000 cGy	4	2823 cGy

For daily treatment delivery, the patient was set up on the couch using the surface-guided radiotherapy (SGRT) system of AlignRT™ (Vision RT Ltd., London, UK) and translated to the treatment isocenter. A CBCT scan was acquired, followed by rigid registration with the planning scan and PET CT. Organs at risk (OAR) and primary targets were auto-generated by the AI in a two-step process, reviewed, and edited by the radiation oncologist. Two plans were provided by the system, scheduled and adapted, and one was selected for that day’s treatment. The adapted plan was chosen for all fractions. In our case, better PTV coverage was seen in 26 of the 33 fractions with the adapted plan. The mean GTV coverage was 99.49% in scheduled plans compared to 99.93%, although not statistically significant. However, statistically significant (p < 0.0.5) coverage was seen with adapted plans in terms of the PTV coverage, Dmax, lungs V20, and esophageal Dmean and Dmax (Table 2). All days with lesser coverage in adapted plans demonstrated improvement in the hotspot reduction and reduction in dose constraints of the esophagus and lungs (Table 3). On the seven days where scheduled plan coverage was better, the mean GTV and PTV coverage in the scheduled plan was 99.93% and 88.31%, respectively, whereas in the adapted plan, it was 99.87% and 87%, respectively. For these minor changes in the coverage, the range of Dmax was 107.1-113.1% in scheduled versus 101.8-105.4% in adapted plans. The esophageal Dmax was 183-192 cGy in scheduled versus 179-183 cGy in adapted plans (Table 4). Overall, adapted plan coverage was 3.91% more than the scheduled plan, with a 6.84% reduction in Dmax, 4.21% and 4.57% reduction in esophageal Dmean and Dmax, and 9.56% reduction in V20 of lungs. To summarize, adapted plans improved coverage in 26 fractions, and on days where coverage was equivalent or less, adapted plans showed drastic improvement in dose avoidance to critical structures.

**Table 2:** Comparison of scheduled plan with adapted plan This table highlights the statistically significant superiority of the adaptive plans over the scheduled plans in comparing the coverage and dose constraints of the esophagus and lungs. GTV, gross tumor volume; PTV, planning target volume; V95, volume receiving 95% of the dose; Dmax, maximum point dose; Dmean, mean dose; V20, volume receiving 2000 cGy; SD, standard deviation

Structure	Scheduled		Adapted		p-value
	Mean	SD	Mean	SD	
GTV V95	99.49	1.41	99.93	0.17	0.112
PTV V95	84.79	2.96	87.45	1.06	<0.001
Dmax	111.39	3.14	103.77	0.81	<0.001
Esophagus Dmean	74.37	4.64	71.23	5.22	<0.001
Esophagus Dmax	189.07	5.45	180.43	1.04	<0.001
Lungs V20	8.48	0.26	7.67	0.89	<0.001

**Table 3:** Data of days (fraction number) where scheduled plan coverage was better This table details the dosimetric evaluation of scheduled and adapted plans on the days when target coverage was better with scheduled plans. It demonstrates better sparing of organs at risk with adapted plans. GTV, gross tumor volume; PTV, planning target volume; V95, volume receiving 95% of the dose; Dmax, maximum point dose; Dmean, mean dose; V20, volume receiving 2000 cGy; SP, scheduled plan; AP, adapted plan

Fraction number	GTV V95		PTV V95		Dmax		Esophagus Dmean		Esophagus Dmax		Lungs V20	
	SP	AP	SP	AP	SP	AP	SP	AP	SP	AP	SP	AP
1	99.8	99.5	87.9	85.2	107.1	102.8	72	67	184	181	8.6	6.3
9	100	99.7	88.1	86.2	107.2	101.8	78	76	184	179	8.4	7.2
13	100	100	88.5	87.1	110.5	104.2	72	70	183	180	8.3	6.5
18	100	99.9	89.5	87.6	113.1	104.1	67	62	184	180	8.6	7
22	99.7	100	88.1	87.4	110.9	104.2	81	78	195	180	8.2	7.4
25	100	100	88.6	88.5	111.6	105.2	73	69	185	180	8.2	6.5
30	100	100	87.5	87	112.4	105.4	74	71	192	183	8	6.9

**Table 4:** Data comparing clinical goals on days where scheduled plan coverage was better This table demonstrates improved dosimetry of the esophagus and lungs with adapted plans, while similar target coverage is seen in both scheduled and adapted plans. GTV, gross tumor volume; PTV, planning target volume; V95, volume receiving 95% of the dose; Dmax, maximum point dose; Dmean, mean dose; V20, volume receiving 2000 cGy

Structure	Scheduled	Adapted
Mean		
GTV V95	99.93%	99.87%
PTV V95	88.31%	87.00%
Esophagus Dmean	73.86 cGy	70.43 cGy
Lungs V20	8.33%	6.83%
Range		
Dmax	107.1-113.1 cGy	101.8-105.4 cGy
Esophagus Dmax	183-192 cGy	179-183 cGy

The patient completed the course in January 2024 without requiring any treatment breaks. She experienced mild odynophagia, which was relieved with oral analgesics. She tolerated the treatment well and did not develop any other toxicities.

**Figure 8:**
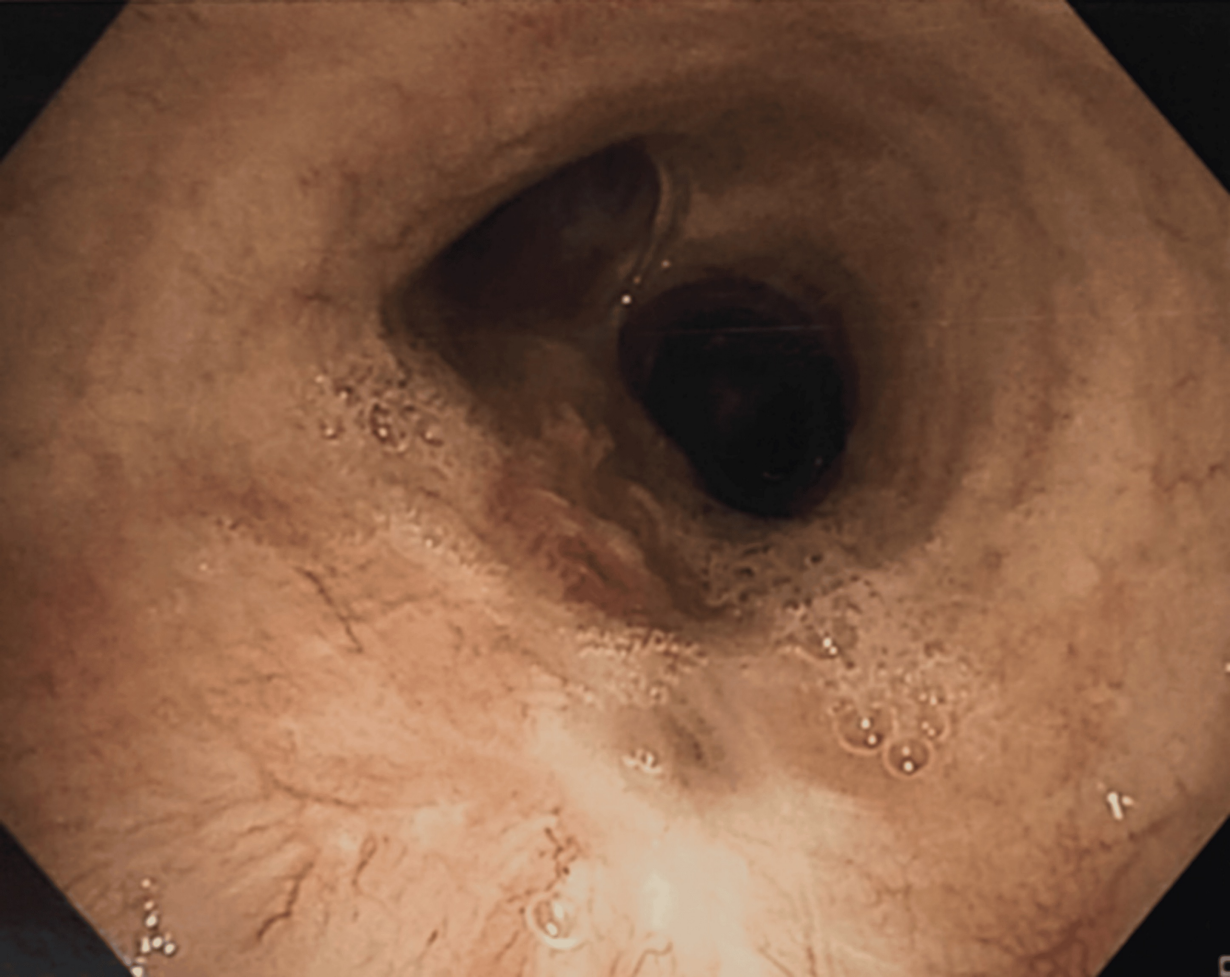
Bronchoscopy It shows smooth tracheal mucosa with a scar at the distal end of the trachea.

Two months after treatment, a follow-up contrast-enhanced CT chest did not reveal any wall thickening of the trachea, although some sequelae of the previously treated tuberculosis were visualized. Follow-up bronchoscopy in July 2024 did not reveal any endobronchial growth in the trachea or either main bronchus; it had smooth mucosa and scar tissue at the distal end of the trachea, suggesting a complete response to radiotherapy (Figure 8). The patient is currently alive and asymptomatic.

## Discussion

The incidence rate of primary tracheal carcinoma is 0.1-0.26 per 1,00,000 population, accounting for 0.1-0.4% of malignant diseases. Adult tracheal tumors are rare, and 80-90% are malignant, according to the literature review. Tracheal ACC occurs in the fourth and fifth decades of life. It has equal predilection in both sexes [6].

Squamous cell carcinoma (SCC) originates predominantly in the distal trachea, whereas ACC originates in the proximal trachea [6]. However, the location of tumor origin varies between various studies. In our case, the tumor is in the distal trachea abutting the carina.

It has a long clinical course with low malignant potential, leading to local recurrence and late-onset metastasis. Local spread is through direct submucosal extension or along perineural planes, whereas distant metastasis occurs through a hematogenous route [6]. Regional lymph node or distant metastasis at presentation is seen in 10% of the cases due to the delicate arterial and lymphatic network. The lung is the most common site for distant metastasis [7].

Radical surgical resection is found to have better survival outcomes in tracheal ACC. The reported five-year survival rate is 88-100%, and the 10-year survival rate is 51-80% with surgery alone [8,9]. However, with higher submucosal spread rates in tracheal ACC and the inability to achieve clear margins, complete resection is not usually achievable, and the percentage of positive resection margin is 59-63%. In such cases, attempting complete excision of the tumor results in higher surgical complications and mortality rates of 1-25% [8-10]. The indications for adjuvant radiotherapy are positive or close margin, microscopic or gross residual disease, perineural invasion, and the presence of positive lymph nodes [11], leading to better local control and improved disease-free survival rates [12]. However, some trials do not reflect survival benefits despite a benefit in locoregional control [13]. Radiotherapy alone or chemoradiotherapy is recommended in inoperable cases, but its effectiveness is not clear. Högerle et al. [14] reported that the curative effect of definitive radiotherapy is comparable to surgery followed by adjuvant radiotherapy. Similarly, in our case, although the patient had a stable response to chemotherapy on a CT scan, bronchoscopy revealed a 5.5 cm long submucosal disease that was deemed unresectable according to Grillo and Mathisen’s criteria of the tumor reaching up to the carina and the length of resection exceeding 6 cm [15], hence had to be addressed with local definitive radiotherapy.

Due to the lack of an optimal dose and fractionation schedule, radiation therapy in tracheal ACC is individualized based on the extent of resection, status of positive margins, and patient-specific considerations. In radical radiotherapy, doses in the range of 46-80 Gy have been utilized, with the median dose being 60 Gy based on a systematic review of 1252 cases [16]. Most studies have reported the use of a 60-70 Gy radiation dose to be sufficient for definitive treatment with or without concurrent chemotherapy, demonstrating equivalent survival outcomes as compared to surgery with or without adjuvant radiotherapy [17-19], although one by Bhandari et al. used a total definitive dose of 54 Gy only [20]. One study did demonstrate significantly improved overall survival (OS) with dose escalation beyond 60 Gy with high dose rate (HDR) brachytherapy doses of 15-21 Gy in three fractions; however, they reported a higher rate of tracheal stenosis [5]. Maximum cases in these studies were treated using the three-dimensional conformal radiotherapy (3DCRT) technique as opposed to IMRT with o-ART in our case. We treated our patient in two phases: a larger volume covering potential microscopic disease to a dose of 54 Gy in 30 fractions and a boost phase of 5.4 Gy in three fractions.

To the best of our knowledge, this is the first case demonstrating the use of o-ART technology with SGRT for definitive radiotherapy of a rare malignancy like tracheal ACC. The trachea is a mobile structure, so o-ART enabled us to account for its inter-fractional positional variability and accurately delineate the GTV on the daily CBCT scan to generate appropriate PTV, which ensured adequate coverage and prevented any target miss. We could also delineate the esophagus, heart, and lungs daily to accurately record the dose received by these organs and correlate clinically. With o-ART, we could reduce the margins considerably compared to the other studies. A retrospective study of 31 patients with tracheal ACC utilized a 4-5 cm expansion from GTV to CTV [4], a study with 22 patients assessing 10-year clinical outcomes in tracheal ACC utilized GTV to CTV expansion of 3 cm longitudinally followed by a 1 cm PTV margin for the entire course [5], and a study of 32 patients assessing all tracheal carcinomas (10 of whom were ACC) had a 3-5 cm longitudinal and 1-2 cm axial CTV expansion along with another 1 cm PTV expansion [19]. In contrast to these studies, our study reduced the GTV to CTV expansion to 1 cm, phase I PTV margin to 5 mm, and phase II PTV margin to 2 mm with the adaptive process. This reduced the OAR doses, which reflected clinically in our patient, who experienced mild odynophagia alone compared to grade 2 and 3 esophagitis, dysphonia, and mucositis in the retrospective studies [4,5].

## Conclusions

We report the first case of tracheal ACC treated with Ethos-based o-ART technology assisted with SGRT. Our results highlight the superior target coverage and improved OAR sparing plans in daily o-ART compared to IGRT plans. The system’s ability to account for daily anatomical changes and provide improved target coverage with better OAR sparing makes it an encouraging option for malignancies requiring motion management.
